# Dopaminergic and Metabolic Correlations With Cognitive Domains in Non-demented Parkinson’s Disease

**DOI:** 10.3389/fnagi.2021.627356

**Published:** 2021-02-16

**Authors:** Linlin Han, Jiaying Lu, Yilin Tang, Yun Fan, Qisi Chen, Ling Li, Fengtao Liu, Jian Wang, Chuantao Zuo, Jue Zhao

**Affiliations:** ^1^Department of Neurology and National Clinical Research Center for Aging and Medicine, Huashan Hospital, Fudan University, Shanghai, China; ^2^PET Center, Huashan Hospital, Fudan University, Shanghai, China

**Keywords:** Parkinson’s disease, cognitive domain, mild cognitive impairment, dopamine transporter, glucose metabolism

## Abstract

**Background:**

Accruing positron emission tomography (PET) studies have suggested that dopaminergic functioning and metabolic changes are correlated with cognitive dysfunction in Parkinson’s disease (PD). Yet, the relationship between dopaminergic or cerebral metabolism and different cognitive domains in PD is poorly understood. To address this scarcity, we aimed to investigate the interactions among dopaminergic bindings, metabolic network changes, and the cognitive domains in PD patients.

**Methods:**

We recruited 41 PD patients, including PD patients with no cognitive impairment (PD-NC; *n* = 21) and those with mild cognitive impairment (PD-MCI; *n* = 20). All patients underwent clinical evaluations and a schedule of neuropsychological tests and underwent both 11C-N-2-carbomethoxy-3-(4-fluorophenyl)-tropane (11C-CFT) and 18F-fluorodeoxyglucose (18F-FDG) PET imaging.

**Results:**

11C-CFT imaging revealed a significant positive correlation between executive function and striatal dopamine transporter (DAT) binding at both the voxel and regional levels. Metabolic imaging revealed that executive function correlated with 18F-FDG uptake, mainly in inferior frontal gyrus, putamen, and insula. Further analysis indicated that striatal DAT binding correlated strictly with metabolic activity in the temporal gyrus, medial frontal gyrus, and cingulate gyrus.

**Conclusion:**

Our findings might promote the understanding of the neurobiological mechanisms underlying cognitive impairment in PD.

## Introduction

Cognitive decline is a prevalent comorbidity in Parkinson’s disease (PD), and up to 80% of patients ultimately suffer from dementia (PDD) (Hely et al., 2008). Mild cognitive impairment in PD (PD-MCI) represents a less severe cognitive deficit in patients and is defined as a transition from unnoticeable changes in cognition to dementia (Kehagia et al., 2010).

The pathophysiological mechanisms underlying PD-MCI are not well understood, although a wide variety of neurotransmitter deficits, including dopamine, acetylcholine, and norepinephrine deficits, have been proven to contribute (Halliday et al., 2014). Neuroimaging has considerably improved the understanding of the pathophysiological basis underlying cognitive impairment. Cognitive impairment in PD is characterized by deficits in major cognitive domains including executive function, attention, memory, visuospatial function, and language (Foltynie et al., 2004; Papagno and Trojano, 2018). However, detailed investigations of the relationship between neuroimaging and different cognitive domains in PD are limited. Some positron emission tomography (PET) studies have demonstrated the significant correlation between striatal dopamine transporter (DAT) binding and executive function (Rinne et al., 2000; Nobili et al., 2010; Siepel et al., 2014; Pellecchia et al., 2015; Kim et al., 2019), whereas a recent study found that DAT binding in the caudate was also well correlated with memory and visuospatial dysfunction (Chung et al., 2018).

Parkinson’s disease-related cognitive deficits go beyond the dopamine system. Accordingly, to explore metabolic network changes at a system level, ^18^F-fluorodeoxyglucose (^18^F-FDG) PET has been utilized. Our previous studies reported different cerebral glucose metabolisms among PDD patients, PD-MCI patients, and PD patients with normal cognition (PD-NC) (Tang et al., 2016). We suggest that all cognitive domains except language are associated with cerebral metabolism, mainly in the posterior cortical areas (Wu et al., 2018).

Nonetheless, information is scant concerning either dopaminergic integrity or whole-brain metabolic changes alone. Thus, the combined assessment of dopaminergic and metabolic imaging has been explored in few studies (Polito et al., 2012; Niethammer et al., 2013; Kim et al., 2019). However, these studies did not directly evaluate the interactions between cognitive domains and PET imaging. Herein, we conducted a dual-tracer PET study, with both ^11^C-N-2-carbomethoxy-3-(4-fluorophenyl)-tropane (^11^C-CFT) and ^18^F-FDG PET imaging, in a cohort of 20 PD-MCI and 21 PD-NC patients. We aimed to investigate the interactions among dopaminergic abnormalities, cerebral metabolism, and the different cognitive domains and gain further insights into the neurobiological mechanisms related to cognitive impairment in PD.

## Materials and Methods

### Subjects

A total of 41 PD patients aged 50–80 years were consecutively enrolled from February 2012 to November 2016 in the Department of Neurology, Huashan Hospital affiliated with Fudan University. The patients were diagnosed by at least two specialists on movement disorders based on the United Kingdom PD Society Brain Bank (Hughes et al., 1992). Patients were excluded from the analysis if: (a) had a diagnosis of dementia (Emre et al., 2007) at baseline; (b) had undergone deep brain stimulation; (c) had major psychiatric disorder; (d) had a history of stroke and/or head injury; and (e) previous genetic testing related to PD. Both ^11^C-CFT and ^18^F-FDG PET were conducted in the same individuals.

The study was approved by the Human Studies Institutional Review Board of Huashan Hospital (Approval No.: 2011-174-3), and informed written consent based on the Declaration of Helsinki guidelines was provided by all enrolled patients.

### Clinical and Cognitive Assessments

Clinical evaluations were conducted at Huashan Hospital. No anti-parkinsonian medications were administered to the patients no less than 12 h prior to the assessments. The modified Hoehn and Yahr scale and the Unified Parkinson’s Disease Rating Scale motor (UPDRS-III) sub-score were used to assess the stage and severity of parkinsonism for each patient. And the Geriatric Depression Rating Scale (GDS) was used to evaluate depression (Yesavage et al., 1982). The dosage of anti-parkinsonian drugs was converted into a total daily levodopa equivalent dose (LED) to standardize the medication data (Schade et al., 2020).

After the motor assessment, patients took the cognitive assessment in the ON condition. The Mini Mental State Examination (MMSE) was performed to assess global cognitive function (Katzman et al., 1988) and a full set of neuropsychological tests for five specific cognitive domains were carried out as follows: (1) attention and working memory: the Symbol Digit Modality Test (SDMT) (Sheridan et al., 2006) and Trail Making Test A (TMT-A) (Zhao et al., 2013); (2) executive function: Stroop Color-Word Test (CWT) (Steinberg et al., 2005) and Trail Making Test B (TMT-B) (Zhao et al., 2013); (3) language: Boston Naming Test (BNT) and Animal Fluency Test (AFT) (Lucas et al., 2005); (4) memory: Auditory Verbal Learning Test (AVLT) (Guo et al., 2009) and delayed recall of the Rey-Osterrieth Complex Figure Test (Caffarra et al., 2002); and (5) visuospatial function: Clock Drawing Test (Ricci et al., 2016) and copy task of Rey-Osterrieth Complex Figure test (Caffarra et al., 2002).

To obtain normative data for the Chinese adult population, 100 healthy subjects matched for age, education, and sex were recruited as controls (Supplementary Table 1). We transformed the raw score of individual neuropsychological tests into Z-scores by subtracting the mean test score of the control sample from an individual raw score and then dividing the difference by the standard deviation of the controls using the following formula: *Z*−*Score* = (*test score*−*Mean*_*control*_)÷*SD*_*control*_. The mean of two or three Z-scores of the same domain was calculated as the Z-score for each cognition domain.

The MDS Task Force Level 2 was used to determine a PD-MCI diagnosis (Litvan et al., 2012). A score 1.5 SDs below the norm for a given cognitive test was defined as abnormal. PD-MCI was diagnosed based on the detection of impairment on two or more neuropsychological tests, characterized by either one impaired test involving two independent cognitive domains or two impaired tests involving the same domain.

### PET Imaging

One week before or after the neuropsychological assessments, all patients underwent ^11^C-CFT PET and then underwent ^18^F-FDG PET on the following day. No anti-parkinsonian medications were administered to the patients within 12 h prior to PET imaging. To prepare for ^18^F-FDG PET, subjects were additionally required to fast for at least 6 h before the PET scan. The patients underwent a Siemens Biograph 64 PET/CT (Siemens, Munich, Germany) in 3D mode. A low-dose CT transmission scan was performed for attenuation correction. Then, PET scanning was acquired during the interval of 60–75 min after 350–400 MBq of ^11^C-CFT was intravenously injected. For ^18^F-FDG, a 10-min PET scan was acquired at 45 min post injection (150–200 MBq). Image reconstruction was obtained by the ordered subset expectation maximization 3D (OSEM 3D) method. All patients were placed in a quiet, dimly lit room to rest.

### Imaging Processing and Data Analysis

Experienced physicians from the nuclear medicine clinic analyzed all images without knowing the patient’s clinical diagnosis. SPM8 software (Wellcome Department of Imaging Neuroscience, Institute of Neurology, London, United Kingdom) implemented in MATLAB 8.4 (MathWorks Inc., Sherborn, MA, United States) was used for data pre-processing, and ScAnVP software Version 7.1.0 (Center for Neuroscience, The Feinstein Institute for Medical Research, Manhasset, NY, United States) was subsequently used for data processing. All images were spatially normalized into Montreal Neurological Institute (MNI) brain space. A brain DAT binding template in MNI space that was created by using ^11^C-CFT PET and corresponding structural MR images of another group consisting of 16 normal controls was used to normalize the ^11^C-CFT images. The procedures were presented in detail in former studies (Ma et al., 2010; Bu et al., 2018). After normalization, the PET images were then smoothed with an isotropic Gaussian kernel of 10 mm (^18^F-FDG) or 8 mm (^11^C-CFT) at full-width at half-maximum.

The regional binding of ^11^C-CFT in the striatum was quantified as the standard uptake value ratios (SUVRs) in the caudate nucleus, the anterior putamen (APU), and the posterior putamen (PPU). The detailed procedure was described previously (Bu et al., 2018). To calculate the regional DAT bindings, we placed the standard regions of interest (ROIs) for the caudate nucleus, APU, and PPU along the longitudinal axis and for the occipital cortex on the mean image summed over the central striatal slices and then adjusted each individually. Estimated hemispherically by the striatal-to-occipital ratio (SOR), defined as (striatum-occipital)/occipital counts, the regional DAT bindings were calculated in succession to obtain the average amount across hemispheres (Huang et al., 2020).

To obtain the SUVR maps from the ^11^C-CFT data, the smoothed images were individually calculated by the occipital counts as the reference [image/occipital counts-1] (Liu et al., 2018).

### Statistical Analysis

A Student *t*-test and Mann–Whitney *U*-test were used to analyze the demographic profiles and neuropsychological characteristics at baseline in the PD-MCI and PD-NC patients, as appropriate.

#### Correlations Between Maps of DAT Bindings or Striatal DAT Bindings and Z-Scores of Cognitive Domains

To investigate the relationships between maps of DAT bindings and Z-scores of cognitive domains, multiple regression analyses in SPM8 were performed (Gratwicke et al., 2013). The threshold was set at *P* < 0.001 (uncorrected) for significant regions. Talairach Daemon software (Research Imaging Center, University of Texas Health Science Center, San Antonio, TX, United States) was employed to orient the significant regions after accomplishing an MNI-to-Talairach conversion. SPM t-maps were overlaid on a standard T1-weighted MRI brain template in stereotaxic space. A 4-mm radius spherical volume of interest (VOI) in the image space, centering at the peak voxel of clusters that were significant in each SPM analysis, was constructed to extract the specific DAT bindings using the ScAnVP software mentioned above. Then, the specific DAT bindings were correlated with the cognitive scores by Pearson’s correlation using SPSS software. *P* < 0.05 was considered significant.

In addition, the correlations between striatal DAT bindings (average ^11^C-CFT uptakes in caudate, APU, and PPU) and Z-scores of cognitive domains were tested separately in all patients by Pearson’s correlation in SPSS software.

#### Correlations Between Global Glucose Metabolism and Z-Scores of Cognitive Domains

We conducted multiple regression analyses to explore the relevance among global glucose metabolism and Z-scores of cognitive domains (Liu et al., 2018). Mean signal differences over the whole brain were removed by analysis of covariance per subject. The voxel threshold was set at *P* < 0.001 (uncorrected) over the whole brain for significant correlation evaluation. Significant region localization and SPM t-map overlying were accomplished using the methods described above. To explore *post hoc* correlations of imaging measures with cognitive scores in patients, we extracted the corresponding VOI values of ^18^F-FDG images using global counts [regional cerebral metabolic rate of glucose (rMRglc)] with ScAnVP software described previously. The correlations were estimated by Pearson’s correlation in SPSS.

#### Correlations Between Global Glucose Metabolism and Striatal DAT Bindings

To explore whether there was a potential association in cognitive function between these two different metabolic activities, multiple regression analyses were conducted to investigate the relationship among global glucose metabolism and average ^11^C-CFT uptakes in caudate, APU, and PPU (Liu et al., 2018). UPDRS-III sub-scores were used as covariates to eliminate confounding factors. Mean signal differences over the whole brain were removed by analysis of covariance per subject. The voxel threshold was set at *P* < 0.001 (uncorrected) over the whole brain for significant correlation evaluation. Significant region localization and SPM t-map overlying were accomplished using the methods described above. Similarly, the rMRglcs in the corresponding peak points were extracted with ScAnVP software described previously for *post hoc* analysis. Partial correlations were applied for these analyses with UPDRS-III sub-scores as covariate in SPSS.

## Results

### Clinical Characteristics and Cognitive Profiles in PD Patients

A total of 41 PD patients including 20 PD-MCI and 21 PD-NC patients were enrolled in this study. No group differences were found for age, sex, education, disease severity (duration, H&Y stage, and UPDRS-III sub-score), LED, or GDS score. The PD-MCI group showed worse performance in global cognitive function (MMSE, *P* = 0.028) compared to that in the PD-NC group. In detail, Z-scores of executive function, attention, memory, and visuospatial function in the PD-MCI group were poorer than those in the PD-NC group (*P* < 0.05), while Z-score of language was relatively preserved (Table 1).

**TABLE 1:** Clinical characteristics and cognitive profile in PD patients.

	PD-NC	PD-MCI	*P*-values
No.	21	20	/
Age (year)	60.43 ± 7.30	62.40 ± 7.39	0.407
Gender (male/female)	15/6	13/7	0.658
Education (year)	13.20 ± 2.84	12.10 ± 3.82	0.285
Disease duration (month)	33.23 ± 23.50	50.00 ± 54.70	0.218
UPDRS-III subscore	23.19 ± 11.49	25.82 ± 17.99	0.605
Hoehn and Yahr stage (IQR)	2 (2–3)	2 (2–3)	0.081
LED (mg/day)	297.09 ± 181.99	468.56 ± 459.08	0.168
GDS score	9.26 ± 7.01	9.33 ± 5.48	0.973
MMSE	28.52 ± 1.21	27.40 ± 1.85	0.028*
Executive function (Z-score)	−0.08 ± 0.60	−0.61 ± 0.77	0.017*
CWT-C time (s)	71.38 ± 25.46	74.90 ± 34.18	0.710
CWT-C right	46.62 ± 4.55	44.05 ± 6.20	0.137
TMT-B (s)	146.67 ± 37.00	162.84 ± 52.95	0.266
Attention (Z-score)	−0.01 ± 0.51	−0.50 ± 0.71	0.015*
SDMT	40.62 ± 10.14	47.75 ± 36.89	0.433
TMT-A (s)	53.90 ± 14.65	64.70 ± 26.27	0.117
Language (Z-score)	0.05 ± 0.92	−0.55 ± 0.98	0.05
BNT	25.95 ± 5.47	26.00 ± 9.80	0.985
AFT	17.95 ± 6.09	14.30 ± 7.64	0.098
Memory (Z-score)	−0.36 ± 1.02	−1.24 ± 1.13	0.012*
AVLT-delay recall	6.67 ± 7.11	9.50 ± 13.98	0.424
AVLT-T	26.38 ± 8.23	19.75 ± 9.61	0.022*
CFT-delay recall	15.90 ± 6.91	10.82 ± 7.32	0.037*
Visuospatial function (Z-score)	0.81 ± 4.10	−1.42 ± 2.41	0.041*
CFT	36.00 ± 13.44	30.00 ± 5.38	0.071
CDT	21.7 ± 6.03	16.55 ± 9.21	0.044*

Data are given as mean ± standard deviation (SD) values.* P < 0.05.PD-MCI, Parkinson’s disease with mild cognitive impairment; PD-NC, Parkinson’s disease with normal cognition; H&Y, Hoehn and Yahr stage; IQR, interquartile range; MMSE, Mini Mental State Examination; CWT, Stroop Color-Word Test; TMT, Trail Making Test; SDMT, Symbol Digit Modality Test; BNT, Boston Naming Test; AFT, Animal Fluency Test; AVLT, Auditory Verbal Learning Test; CFT, the Rey-Osterrieth Complex Figure Test; CFT, Clock Drawing Test.

### Correlations Between DAT Bindings and Cognitive Domains

**FIGURE 1:**
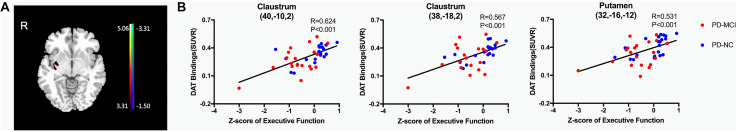
Brain regions exhibiting a significant correlation between Z-score of executive function and regional DAT bindings. **(A)** Correlations between the Z-score of executive function and 11C-CFT uptake in PD patients. Positive correlations are displayed using a red–yellow scale and negative correlations using a blue–green scale. The overlays are depicted in neurologic orientation. The gray-scale image is the standard T1-weighted structural magnetic resonance image (MRI) in Montreal Neurological Institute (MNI) space. The thresholds of the color bars represent T values. Voxel threshold was set at *P* < 0.001. **(B)** In *post hoc* analysis, correlations of Z-score of executive function with regional DAT bindings are shown. DAT, dopamine transporter; PD-MCI, Parkinson’s disease with mild cognitive impairment; PD-NC, Parkinson’s disease with normal cognition.

At the regional level, the average DAT bindings in APU and PPU showed positive correlations with the Z-score of executive function (APU: *P* = 0.049, *R* = 0.308; PPU: *P* = 0.033, *R* = 0.334), though the one in the caudate only showed a broadly similar correlation, albeit without reaching significance (*P* = 0.061, *R* = 0.296). However, no significant correlation with other cognitive domains was observed (Supplementary Table 2). The voxel-wise analysis showed similar results. The Z-score of executive function correlated positively with DAT bindings in the claustrum and putamen (*P* < 0.001, uncorrected) (Figure 1A and Table 2). The specific bindings in these regions also showed positive correlations with the Z-score of executive function (claustrum: *P* < 0.001, *R* = 0.624; claustrum: *P* < 0.001, *R* = 0.567; putamen: *P* < 0.001, *R* = 0.531) (Figure 1B).

**TABLE 2:** Brain regions exhibiting a significant correlation between Z-score of executive function and regional DAT bindings.

Region†	Hemi-sphere	Cluster size (mm^3^)	T Max	Z Max	Coordinates*		
					X	Y	Z
**Positive**							
Claustrum††	Right	1880	5.06	4.41	40	−10	2
Claustrum††	Right		4.27	3.84	38	−18	2
Lentiform nucleus (putamen) ††	Right		3.87	3.54	32	−16	−12

*Coordinates are displayed in MNI standard space.†Significant at voxel threshold of P < 0.001 (uncorrected), extent threshold = 228 voxels (1824 mm^3^).††The cluster also survived at family-wise error-corrected P < 0.05.BA, Brodmann area.

### Correlations Between Global Glucose Metabolism and Cognitive Domains

**FIGURE 2:**
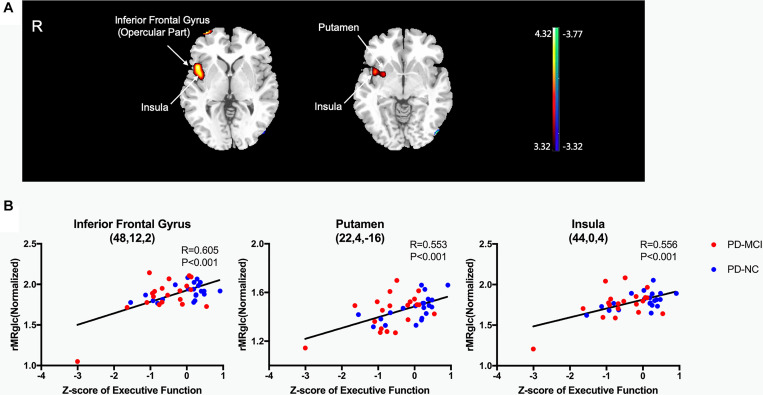
Brain regions exhibiting a significant correlation between Z-score of executive function and 18F-FDG uptake. **(A)** Correlations between the 18F-FDG uptake and Z-score of executive function in PD patients. Positive correlations are displayed using a red–yellow scale and negative correlations using a blue–green scale. The overlays are depicted in neurologic orientation. The gray-scale image is the standard T1-weighted structural magnetic resonance image (MRI) in Montreal Neurological Institute (MNI) space. The thresholds of the color bars represent T values. Voxel threshold was set at *P* < 0.001. **(B)** In *post hoc* analysis, correlations of Z-score of executive function with regional glucose metabolic activities are shown. rMRglc, regional cerebral metabolic rate of glucose; PD-MCI, Parkinson’s disease with mild cognitive impairment; PD-NC, Parkinson’s disease with normal cognition.

Only executive function showed significant correlation with glucose metabolism in voxel-wise analyses (*P* < 0.001, uncorrected). The Z-scores of executive function correlated positively with ^18^F-FDG metabolism in the inferior frontal gyrus (opercular part), putamen, and insula (Table 3 and Figure 2A). No negative correlation was observed. In *post hoc* analyses, the values of the normalized rMRglc in these specific regions showed consistent results (Figure 2B).

**TABLE 3:** Brain regions exhibiting significant correlations between Z-scores of executive function and regional brain glucose metabolism.

Region†	Hemi-sphere	Cluster size (mm^3^)	T Max	Z Max	Coordinates*		
					X	Y	Z
**Positive**							
*Executive function*							
Inferior frontal gyrus (opercular part)††	Right	5816		3.85	48	18	2
Lentiform nucleus (putamen)††	Right			3.73	22	4	−16
Insual (BA 13)††	Right			3.69	44	0	4

*Coordinates are displayed in MNI standard space.†Significant at voxel threshold of P < 0.001 (uncorrected), extent threshold = 110 voxels (880 mm^3^).††The cluster also survived at family wise-error corrected P < 0.05.BA, Brodmann area.

### Correlations Between Striatal DAT Binding and Whole-Brain Glucose Metabolism

**FIGURE 3:**
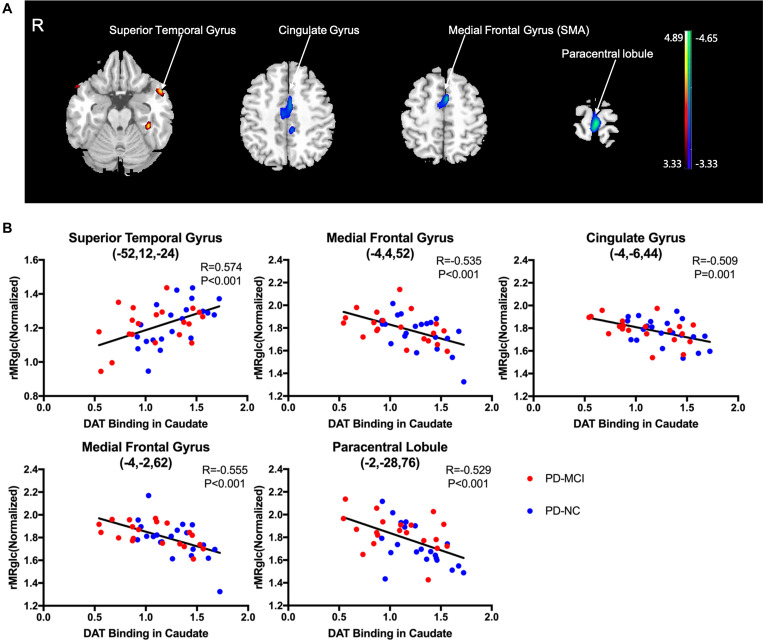
Brain regions exhibiting a significant correlation between average DAT binding in caudate and 18F-FDG uptake. **(A)** Correlations between the average DAT binding in caudate and 18F-FDG uptake in PD patients. Positive correlations are displayed using a red–yellow scale and negative correlations using a blue–green scale. The overlays are depicted in neurologic orientation. The gray-scale image is the standard T1-weighted structural magnetic resonance image (MRI) in Montreal Neurological Institute (MNI) space. The thresholds of the color bars represent T values. Voxel threshold was set at *P* < 0.01. **(B)** In *post hoc* analysis, correlations of relative metabolic values for the significantly correlated regions in SPM and average DAT binding in caudate are shown. rMRglc, regional cerebral metabolic rate of glucose; DAT, dopamine transporter; PD-MCI, Parkinson’s disease with mild cognitive impairment; PD-NC, Parkinson’s disease with normal cognition.

The average DAT binding in the caudate correlated positively with ^18^F-FDG uptake in the superior temporal gyrus (Figure 3A and Table 4). Inverse correlations were found in the medial frontal gyrus [supplementary motor area (SMA)], cingulate gyrus, and paracentral lobule (Figure 3A and Table 4). The correlations in the APU and PPU were similar (Table 4). In *post hoc* analyses, the values of the normalized rMRglc in these regions all showed significant relationships with striatal DAT bindings (*P* < 0.005) (Figure 3B, only shows the results in caudate).

**TABLE 4:** Brain regions exhibiting significant correlations between the average DAT bindings in sub-region and global glucose metabolism.

Region†	Hemi-sphere	Cluster size (mm^3^)	T Max	Z Max	Coordinates*		
					X	Y	Z
**Positive**							
*Average caudate*							
Superior temporal gyrus (BA 38)	Left	1008	4.51	4.00	−52	12	−24
*Average anterior putamen*							
Superior temporal gyrus (BA 38)	Left	912	4.46	3.96	−52	12	−24
**Negative**							
*Average caudate*							
Medial frontal gyrus (BA6, SMA)††	Left	4329	4.50	3.99	−4	4	52
Cingulate gyrus (BA 24)††			4.17	3.75	−4	−6	64
Medial frontal gyrus (BA6, SMA)††			4.12	3.71	−4	−2	61
Paracentral lobule (BA 4)	Left	1912	4.65	4.10	−2	−28	76
**Average anterior putamen**							
Medial frontal gyrus (BA6, SMA)††	Left	4576	4.63	4.09	−4	4	52
Cingulate gyrus (BA 24)††			4.44	3.95	−4	−10	42
**Average posterior putamen**							
Medial frontal gyrus (BA6, SMA)††	Left	9024	5.18	4.46	−4	0	56
Cingulate gyrus (BA 24)††	Left		4.49	3.98	−6	−8	52
Cingulate gyrus (BA 24)††	Left		4.25	3.81	−4	−42	46

* Coordinates are displayed in MNI standard space.†Significant at voxel threshold of P < 0.001 (uncorrected), extent threshold = 110 voxels (880 mm^3^).††The cluster also survived at family wise-error corrected P < 0.05.BA, Brodmann area; SMA, supplementary motor area.

## Discussion

To our knowledge, this is the first dual-tracer PET study to investigate the relationships among nigrostriatal abnormalities, glucose metabolic changes, and major cognitive domains in PD patients. The main findings were as follows: (1) DAT imaging revealed a significant positive correlation between striatal ^11^C-CFT binding and executive function at both the voxel and regional levels. (2) Glucose metabolic imaging revealed that executive function correlated with ^18^F-FDG uptake in the inferior frontal gyrus, putamen, and insula. (3) Further analysis of dopaminergic imaging and metabolic imaging indicated that DAT binding in the caudate and APU correlated positively with metabolic activity in the temporal gyrus, and negatively with medial frontal and cingulate gyrus.

It is well recognized that nigrostriatal dopaminergic denervation is an important contributor to motor symptoms in PD. In addition, the dopamine system is also involved in cognition, as suggested by accumulating evidence (Grahn et al., 2008). Many studies, mainly evaluating PET data, have revealed that striatal dopamine depletion is related to cognitive impairment in PD (Rinne et al., 2000; Nobili et al., 2010; Siepel et al., 2014; Pellecchia et al., 2015; Chung et al., 2018; Kim et al., 2019). Most of these studies demonstrated a significant association between DAT binding and executive function (Nobili et al., 2010; Siepel et al., 2014; Pellecchia et al., 2015). In support of these results, our study demonstrated a positive correlation between striatal DAT binding and executive performance. ^11^C-CFT uptake in the APU and PPU was shown to be associated with executive function. The APU, which receives projections from the prefrontal cortex, is correlated with working memory (Arsalidou et al., 2013), while the PPU plays a role in the control of habitual actions (Redgrave et al., 2010). These two cognitive abilities are both crucial components of executive function. In addition, the voxel-wise analysis showed executive function correlated positively with DAT bindings in the claustrum. The claustrum is a telencephalic gray matter nucleus which is interconnected with the neocortex. It might subserve frontal cortical function, mediating top-down executive function (White and Mathur, 2018; Krimmel et al., 2019). Our results support that a detailed segmentation of the striatum might provide more information related to neural circuits in cognitive impairment in PD.

The relationship between cognitive domains and cerebral metabolism in PD has yet to be fully elucidated. In our previous study, domains of attention, executive function, memory, and visuospatial function were associated with cerebral metabolism (Wu et al., 2018). However, in the present study, only executive function was found to be associated with cerebral metabolism. The disparity may be explained by the relatively mild cognitive impairment of subjects, without patients with PDD, were enrolled in this study. The executive function was positively correlated with metabolism of inferior frontal gyrus and putamen, which belong to the regions in the fronto-striatal network. Alteration of the fronto-striatal network has been suggested as playing a key role in cognitive dysfunction in PD, especially in executive dysfunction (Owen, 2004). In addition to the inferior frontal gyrus and putamen, our metabolic imaging also revealed that the metabolism of insula was significantly correlated with executive function. The insula functionally belongs to mesocortical network. Besides fronto-striatal network, mesocortical network was also demonstrated to be the neural circuitry underlying executive deficits in PD (Gratwicke et al., 2015). Insular cortex in particular is considered to facilitate cognitive flexibility, a core feature of executive processing (Menon and Uddin, 2010). Christopher et al. suggest that it is supervening dysfunction in the mesocortical projections to the insular upon existing fronto-striatal network disruption that heralds major executive impairment (Christopher et al., 2014).

Another finding is the association between DAT binding and whole-brain metabolism. Reduced DAT binding in the caudate and APU was correlated with reduced metabolism in the superior temporal gyrus and increased metabolism in the medial frontal gyrus and cingulate gyrus. Although future PET studies with specific radioligands need to elucidate the exact specific neurotransmitters contained in the cortical areas such as superior temporal gyrus and cingulate gyrus, this finding might suggest that interaction effects between the dopamine system and other neurotransmitter systems are possibly involved in cognitive impairment in PD. Bohnen et al. used combined [^11^C]PMP acetylcholinesterase and [^11^C]DTBZ monoaminergic PET to investigate the possible interaction effects between dopaminergic and cholinergic systems (Bohnen et al., 2015). The results revealed significant interactive cognitive effects between these two neurotransmitter systems, showing that striatal dopaminergic and cortical cholinergic degenerations contribute to cognitive deficits in PD in both an additive and a synergistic fashion. In an animal study, Kucinski et al. reported that dual dopaminergic-cholinergic lesions in rats induced greater attention deficits than those with only dopaminergic or cholinergic lesions. Another animal study supported the link between the promoted prefrontal cortical GABA level and GABAergic transmission and the amelioration of working memory deficits (Liu et al., 2019). However, how neurotransmitters interact with each other is not well understood. Although multiple neurotransmitter deficits have been observed in underlying cognitive deficits in PD in recent years (Halliday et al., 2014), the interactive cognitive effects between different neurotransmitter systems need to be clarified in future work.

Our study has some limitations. First, this study has a cross-sectional design with a small sample size; hence, dopaminergic-metabolic changes could not be investigated as cognition deteriorated. Thus, further longitudinal studies with a larger group of patients are needed to confirm these results. Furthermore, we limited DAT binding analysis to the nigrostriatal projections without cortex considering the controversial extrastriatal dopaminergic findings of current tracers (Ouchi et al., 2001). Additionally, our study is based on dopaminergic binding and glucose metabolism PET and is limited in its ability to clarify the specific neurotransmitters and the exact neural circuits underlying cognitive deficits in PD. Further studies, ideally with more specific PET tracers, can help to provide better insights in this regard.

## Conclusion

In conclusion, the present study indicated interactions among dopaminergic deficits, cerebral metabolism, and cognitive domains at the voxel and regional levels. Our study might offer a new perspective for the application of dopaminergic-metabolic PET imaging in executive function in PD and promote the understanding of the neurobiological mechanisms underlying cognitive impairment in PD.

## Data Availability Statement

The original contributions presented in the study are included in the article/Supplementary Material. Further inquiries can be directed to the corresponding author/s.

## Ethics Statement

The studies involving human participants were reviewed and approved by the Human Studies Institutional Review Board of Huashan Hospital. The patients/participants provided their written informed consent to participate in this study.

## Author Contributions

LH contributed to conceptualization, methodology, formal analysis, and writing—original draft. JL contributed to formal analysis, data curation, methodology, visualization, and writing—review and editing. YT and CZ contributed to conceptualization, writing—review and editing, and funding acquisition. YF, QC, LL, and FL contributed to data curation and writing—review and editing. JW contributed to funding acquisition and writing—review and editing. JZ contributed to conceptualization, data curation, writing—review and editing, and supervision. All authors contributed to the article and approved the submitted version.

## Conflict of Interest

The authors declare that the research was conducted in the absence of any commercial or financial relationships that could be construed as a potential conflict of interest.

## References

[B1] Arsalidou M. Duerden E. G. Taylor M. J. (2013). The centre of the brain: topographical model of motor, cognitive, affective, and somatosensory functions of the basal ganglia. *Hum. Brain Mapp.* 34 3031–3054. 10.1002/hbm.22124 22711692 PMC6870003

[B2] Bohnen N. I. Albin R. L. Müller M. L. Petrou M. Kotagal V. Koeppe R. A. (2015). Frequency of cholinergic and caudate nucleus dopaminergic deficits across the predemented cognitive spectrum of Parkinson disease and evidence of interaction effects. *JAMA Neurol.* 72 194–200. 10.1001/jamaneurol.2014.2757 25506674 PMC5565160

[B3] Bu L. L. Liu F. T. Jiang C. F. Guo S. S. Yu H. Zuo C. T. (2018). Patterns of dopamine transporter imaging in subtypes of multiple system atrophy. *Acta Neurol. Scand.* 138 170–176. 10.1111/ane.12932 29573392

[B4] Caffarra P. Vezzadini G. Dieci F. Zonato F. Venneri A. (2002). Rey-Osterrieth complex figure: normative values in an Italian population sample. *Neurol. Sci.* 22 443–447. 10.1007/s100720200003 11976975

[B5] Christopher L. Marras C. Duff-Canning S. Koshimori Y. Chen R. Boileau I. (2014). Combined insular and striatal dopamine dysfunction are associated with executive deficits in Parkinson’s disease with mild cognitive impairment. *Brain* 137 565–575. 10.1093/brain/awt337 24334314 PMC4454524

[B6] Chung S. J. Yoo H. S. Oh J. S. Kim J. S. Ye B. S. Sohn Y. H. (2018). Effect of striatal dopamine depletion on cognition in de novo Parkinson’s disease. *Parkinsonism Relat. Disord.* 51 43–48. 10.1016/j.parkreldis.2018.02.048 29526657

[B7] Emre M. Aarsland D. Brown R. Burn D. J. Duyckaerts C. Mizuno Y. (2007). Clinical diagnostic criteria for dementia associated with Parkinson’s disease. *Mov. Disord.* 22 1689–1707;quiz1837. 10.1002/mds.21507 17542011

[B8] Foltynie T. Brayne C. E. Robbins T. W. Barker R. A. (2004). The cognitive ability of an incident cohort of Parkinson’s patients in the UK. The CamPaIGN study. *Brain* 127 550–560. 10.1093/brain/awh067 14691062

[B9] Grahn J. A. Parkinson J. A. Owen A. M. (2008). The cognitive functions of the caudate nucleus. *Prog. Neurobiol.* 86 141–155. 10.1016/j.pneurobio.2008.09.004 18824075

[B10] Gratwicke J. Jahanshahi M. Foltynie T. (2015). Parkinson’s disease dementia: a neural networks perspective. *Brain* 138 1454–1476. 10.1093/brain/awv104 25888551 PMC4614131

[B11] Gratwicke J. Kahan J. Zrinzo L. Hariz M. Limousin P. Foltynie T. (2013). The nucleus basalis of Meynert: a new target for deep brain stimulation in dementia. *Neurosci. Biobehav. Rev.* 37 2676–2688. 10.1016/j.neubiorev.2013.09.003 24035740

[B12] Guo Q. Zhao Q. Chen M. Ding D. Hong Z. (2009). A comparison study of mild cognitive impairment with 3 memory tests among Chinese individuals. *Alzheimer Dis. Assoc. Disord.* 23 253–259. 10.1097/WAD.0b013e3181999e92 19812468

[B13] Halliday G. M. Leverenz J. B. Schneider J. S. Adler C. H. (2014). The neurobiological basis of cognitive impairment in Parkinson’s disease. *Mov. Disord.* 29 634–650. 10.1002/mds.25857 24757112 PMC4049032

[B14] Hely M. A. Reid W. G. Adena M. A. Halliday G. M. Morris J. G. (2008). The Sydney multicenter study of Parkinson’s disease: the inevitability of dementia at 20 years. *Mov. Disord.* 23 837–844. 10.1002/mds.21956 18307261

[B15] Huang Z. Jiang C. Li L. Xu Q. Ge J. Li M. (2020). Correlations between dopaminergic dysfunction and abnormal metabolic network activity in REM sleep behavior disorder. *J. Cereb. Blood Flow Metab.* 40 552–562. 10.1177/0271678X19828916 30741074 PMC7026846

[B16] Hughes A. J. Daniel S. E. Kilford L. Lees A. J. (1992). Accuracy of clinical diagnosis of idiopathic Parkinson’s disease: a clinico-pathological study of 100 cases. *J. Neurol. Neurosurg. Psychiatry* 55 181–184. 10.1136/jnnp.55.3.181 1564476 PMC1014720

[B17] Katzman R. Zhang M. Y. Ouang-Ya-Qu, Wang Z. Y. Liu W. T. Yu E. (1988). A Chinese version of the mini-mental state examination; impact of illiteracy in a Shanghai dementia survey. *J. Clin. Epidemiol.* 41 971–978. 10.1016/0895-4356(88)90034-0 3193141

[B18] Kehagia A. A. Barker R. A. Robbins T. W. (2010). Neuropsychological and clinical heterogeneity of cognitive impairment and dementia in patients with Parkinson’s disease. *Lancet Neurol.* 9 1200–1213. 10.1016/S1474-4422(10)70212-X 20880750

[B19] Kim H. Oh M. Oh J. S. Moon H. Chung S. J. Lee C. S. (2019). Association of striatal dopaminergic neuronal integrity with cognitive dysfunction and cerebral cortical metabolism in Parkinson’s disease with mild cognitive impairment. *Nucl. Med. Commun.* 40 1216–1223. 10.1097/MNM.0000000000001098 31584466

[B20] Krimmel S. R. White M. G. Panicker M. H. Barrett F. S. Mathur B. N. Seminowicz D. A. (2019). Resting state functional connectivity and cognitive task-related activation of the human claustrum. *Neuroimage* 196 59–67. 10.1016/j.neuroimage.2019.03.075 30954711 PMC6629463

[B21] Litvan I. Goldman J. G. Tröster A. I. Schmand B. A. Weintraub D. Petersen R. C. (2012). Diagnostic criteria for mild cognitive impairment in Parkinson’s disease: movement disorder society task force guidelines. *Mov. Disord.* 27 349–356. 10.1002/mds.24893 22275317 PMC3641655

[B22] Liu F. T. Ge J. J. Wu J. J. Wu P. Ma Y. Zuo C. T. (2018). Clinical, dopaminergic, and metabolic correlations in Parkinson disease: a dual-tracer PET study. *Clin. Nucl. Med.* 43 562–571. 10.1097/RLU.0000000000002148 29863572

[B23] Liu Y. Zong X. Huang J. Guan Y. Li Y. Du T. (2019). Ginsenoside Rb1 regulates prefrontal cortical GABAergic transmission in MPTP-treated mice. *Aging* 11 5008–5034. 10.18632/aging.102095 31314744 PMC6682523

[B24] Lucas J. A. Ivnik R. J. Smith G. E. Ferman T. J. Willis F. B. Petersen R. C. (2005). Mayo’s older African Americans normative studies: norms for Boston naming test, controlled oral word association, category fluency, animal naming, token test, WRAT-3 reading, trail making test, stroop test, and judgment of line orientation. *Clin. Neuropsychol.* 19 243–269. 10.1080/13854040590945337 16019707

[B25] Ma Y. Tang C. Chaly T. Greene P. Breeze R. Fahn S. (2010). Dopamine cell implantation in Parkinson’s disease: long-term clinical and (18)F-FDOPA PET outcomes. *J. Nucl. Med.* 51 7–15. 10.2967/jnumed.109.066811 20008998 PMC2946843

[B26] Menon V. Uddin L. Q. (2010). Saliency, switching, attention and control: a network model of insula function. *Brain Struct. Funct.* 214 655–667. 10.1007/s00429-010-0262-0 20512370 PMC2899886

[B27] Niethammer M. Tang C. C. Ma Y. Mattis P. J. Ko J. H. Dhawan V. (2013). Parkinson’s disease cognitive network correlates with caudate dopamine. *Neuroimage* 78 204–209. 10.1016/j.neuroimage.2013.03.070 23578575 PMC3672243

[B28] Nobili F. Campus C. Arnaldi D. De Carli F. Cabassi G. Brugnolo A. (2010). Cognitive-nigrostriatal relationships in de novo, drug-naïve Parkinson’s disease patients: a [I-123]FP-CIT SPECT study. *Mov. Disord.* 25 35–43. 10.1002/mds.22899 20058228

[B29] Ouchi Y. Kanno T. Okada H. Yoshikawa E. Futatsubashi M. Nobezawa S. (2001). Changes in dopamine availability in the nigrostriatal and mesocortical dopaminergic systems by gait in Parkinson’s disease. *Brain* 124(Pt 4) 784–792. 11287377 10.1093/brain/124.4.784

[B30] Owen A. M. (2004). Cognitive dysfunction in Parkinson’s disease: the role of frontostriatal circuitry. *Neuroscientist* 10 525–537. 10.1177/1073858404266776 15534038

[B31] Papagno C. Trojano L. (2018). Cognitive and behavioral disorders in Parkinson’s disease: an update. I: cognitive impairments. *Neurol. Sci.* 39 215–223. 10.1007/s10072-017-3154-8 29043468

[B32] Pellecchia M. T. Picillo M. Santangelo G. Longo K. Moccia M. Erro R. (2015). Cognitive performances and DAT imaging in early Parkinson’s disease with mild cognitive impairment: a preliminary study. *Acta Neurol. Scand.* 131 275–281. 10.1111/ane.12365 25644029

[B33] Polito C. Berti V. Ramat S. Vanzi E. De Cristofaro M. T. Pellicanò G. (2012). Interaction of caudate dopamine depletion and brain metabolic changes with cognitive dysfunction in early Parkinson’s disease. *Neurobiol. Aging* 33:206.e29-39. 10.1016/j.neurobiolaging.2010.09.004 20961661

[B34] Redgrave P. Rodriguez M. Smith Y. Rodriguez-Oroz M. C. Lehericy S. Bergman H. (2010). Goal-directed and habitual control in the basal ganglia: implications for Parkinson’s disease. *Nat. Rev. Neurosci.* 11 760–772. 10.1038/nrn2915 20944662 PMC3124757

[B35] Ricci M. Pigliautile M. D’Ambrosio V. Ercolani S. Bianchini C. Ruggiero C. (2016). The clock drawing test as a screening tool in mild cognitive impairment and very mild dementia: a new brief method of scoring and normative data in the elderly. *Neurol. Sci.* 37 867–873. 10.1007/s10072-016-2480-6 26863871

[B36] Rinne J. O. Portin R. Ruottinen H. Nurmi E. Bergman J. Haaparanta M. (2000). Cognitive impairment and the brain dopaminergic system in Parkinson disease: [18F]fluorodopa positron emission tomographic study. *Arch. Neurol.* 57 470–475. 10.1001/archneur.57.4.470 10768619

[B37] Schade S. Mollenhauer B. Trenkwalder C. (2020). Levodopa equivalent dose conversion factors: an updated proposal including opicapone and safinamide. *Mov. Disord. Clin. Pract.* 7 343–345. 10.1002/mdc3.12921 32258239 PMC7111582

[B38] Sheridan L. K. Fitzgerald H. E. Adams K. M. Nigg J. T. Martel M. M. Puttler L. I. (2006). Normative symbol digit modalities test performance in a community-based sample. *Arch. Clin. Neuropsychol.* 21 23–28. 10.1016/j.acn.2005.07.003 16139470

[B39] Siepel F. J. Brønnick K. S. Booij J. Ravina B. M. Lebedev A. V. Pereira J. B. (2014). Cognitive executive impairment and dopaminergic deficits in de novo Parkinson’s disease. *Mov. Disord.* 29 1802–1808. 10.1002/mds.26051 25284687

[B40] Steinberg B. A. Bieliauskas L. A. Smith G. E. Ivnik R. J. (2005). Mayo’s older americans normative studies: age- and IQ-adjusted norms for the trail-making test, the stroop test, and MAE controlled oral word association test. *Clin. Neuropsychol.* 19 329–377. 10.1080/13854040590945210 16120535

[B41] Tang Y. Ge J. Liu F. Wu P. Guo S. Liu Z. (2016). Cerebral metabolic differences associated with cognitive impairment in Parkinson’s disease. *PLoS One* 11:e0152716. 10.1371/journal.pone.0152716 27064684 PMC4827825

[B42] White M. G. Mathur B. N. (2018). Frontal cortical control of posterior sensory and association cortices through the claustrum. *Brain Struct. Funct.* 223 2999–3006. 10.1007/s00429-018-1661-x 29623428 PMC5995986

[B43] Wu L. Liu F. T. Ge J. J. Zhao J. Tang Y. L. Yu W. B. (2018). Clinical characteristics of cognitive impairment in patients with Parkinson’s disease and its related pattern in 18 F-FDG PET imaging. *Hum. Brain Mapp.* 39 4652–4662. 10.1002/hbm.24311 29999569 PMC6866526

[B44] Yesavage J. A. Brink T. L. Rose T. L. Lum O. Huang V. Adey M. (1982). Development and validation of a geriatric depression screening scale: a preliminary report. *J. Psychiatr. Res.* 17 37–49. 10.1016/0022-3956(82)90033-4 7183759

[B45] Zhao Q. Guo Q. Li F. Zhou Y. Wang B. Hong Z. (2013). The shape trail test: application of a new variant of the trail making test. *PLoS One* 8:e57333. 10.1371/journal.pone.0057333 23437370 PMC3577727

