# Supplementary material for: Dopaminergic and Metabolic Correlations With Cognitive Domains in Non-demented Parkinson’s Disease
Source: Front Aging Neurosci. 2021 Feb 16;13:627356. doi: 10.3389/fnagi.2021.627356 (PMC7921728; doi:10.3389/fnagi.2021.627356)
Supplement: Supplementary file 1 [file Table_1.docx]

Supplementary Material

# Supplementary Figures and Tables

***Supplementary Table 1*:** **Cognitive Profile of Healthy Controls**

| **Cognitive test** | **Number(n)** | **Mean** | **SD** |
| --- | --- | --- | --- |
| **MMSE** | 93.00 | 27.85 | 1.87 |
| **Attention and working memory** | | | |
| SDMT | 97.00 | 34.78 | 15.20 |
| TMT-A (s) | 97.00 | 48.71 | 28.24 |
| **Executive function** | | | |
| CWT-C time (s) | 99.00 | 77.87 | 21.00 |
| CWT-C right | 99.00 | 46.80 | 3.54 |
| TMT-B (s) | 97.00 | 121.37 | 64.24 |
| **Language** | | | |
| BNT | 100.00 | 24.82 | 3.22 |
| AFT | 97.00 | 18.02 | 4.35 |
| **Memory** | | | |
| AVLT-delay recall | 100.00 | 5.93 | 1.41 |
| AVLT-T | 100.00 | 29.54 | 6.21 |
| CFT-delay recall | 100.00 | 16.44 | 5.52 |
| **Visuospatial function** | | | |
| CFT | 100.00 | 33.51 | 3.25 |
| CDT | 100.00 | 25.81 | 6.31 |

Abbreviations: MMSE, Mini Mental State Examination; SDMT, Symbol Digit Modality Test; TMT, Trail Making Test; CWT, Stroop Color-Word Test; BNT, Boston Naming Test; AFT, Animal Fluency Test; AVLT, Auditory Verbal Learning Test; CFT, the Rey-Osterrieth Complex Figure Test; CDT, Clock Drawing Test.

***Supplementary Table 2*. The Relationships Between Regional DAT Bindings and Z-scores of Cognitive Domains in 41 PD Patients**

|  |  | Average Caudate | Average Anterior Putamen | Average Posterior Putamen |
| --- | --- | --- | --- | --- |
| Executive Function | R | 0.296 | 0.308 | 0.334 |
|  | P | 0.061 | 0.049* | 0.033* |
| Attention | R | 0.270 | 0.282 | 0.292 |
|  | P | 0.088 | 0.074 | 0.064 |
| Language | R | 0.163 | 0.214 | 0.218 |
|  | P | 0.309 | 0.180 | 0.170 |
| Memory | R | 0.008 | -0.003 | -0.001 |
|  | P | 0.961 | 0.983 | 0.953 |
| Visuospatial Function | R | 0.223 | 0.157 | 0.179 |
|  | P | 0.126 | 0.327 | 0.264 |

*P<0.05
